# Silymarin: Unveiling its pharmacological spectrum and therapeutic potential in liver diseases—A comprehensive narrative review

**DOI:** 10.1002/fsn3.4010

**Published:** 2024-02-16

**Authors:** Hafiza Madiha Jaffar, Fahad Al‐Asmari, Faima Atta Khan, Muhammad Abdul Rahim, Eliasse Zongo

**Affiliations:** ^1^ University Institute of Diet & Nutritional Sciences, Faculty of Allied Health Sciences The University of Lahore Lahore Pakistan; ^2^ Department of Food and Nutrition Sciences, College of Agricultural and Food Sciences King Faisal University Al‐Ahsa Saudi Arabia; ^3^ Department of Food Science, Faculty of Life Sciences Government College University Faisalabad Pakistan; ^4^ Department of Food Science & Nutrition, Faculty of Medicine and Allied Health Sciences Times Institute Multan Pakistan; ^5^ Laboratoire de Recherche et d'Enseignement en Santé et Biotechnologies Animales Université Nazi BONI Bobo Dioulasso Burkina Faso

**Keywords:** fatty liver disease, milk thistle, non‐alcoholic fatty liver disease, silibinin, silymarin

## Abstract

Liver diseases, encompassing conditions such as cirrhosis, present a substantial global health challenge with diverse etiologies, including viral infections, alcohol consumption, and non‐alcoholic fatty liver disease (NAFLD). The exploration of natural compounds as therapeutic agents has gained traction, notably the herbal remedy milk thistle (*Silybum marianum*), with its active extract, silymarin, demonstrating remarkable antioxidant and hepatoprotective properties in extensive preclinical investigations. It can protect healthy liver cells or those that have not yet sustained permanent damage by reducing oxidative stress and mitigating cytotoxicity. Silymarin, a natural compound with antioxidant properties, anti‐inflammatory effects, and antifibrotic activity, has shown potential in treating liver damage caused by alcohol, NAFLD, drug‐induced toxicity, and viral hepatitis. Legalon® is a top‐rated medication with excellent oral bioavailability, effective absorption, and therapeutic effectiveness. Its active component, silymarin, has antioxidant and hepatoprotective properties, Eurosil 85® also, a commercial product, has lipophilic properties enhanced by special formulation processes. Silymarin, during clinical trials, shows potential improvements in liver function, reduced mortality rates, and alleviation of symptoms across various liver disorders, with safety assessments showing low adverse effects. Overall, silymarin emerges as a promising natural compound with multifaceted hepatoprotective properties and therapeutic potential in liver diseases.

## Graphical abstract

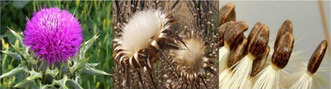

## 1 INTRODUCTION

**FIGURE 1:**
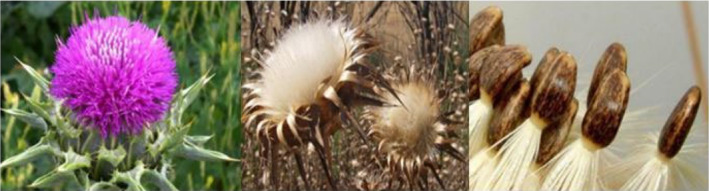
Common names: Milk thistle, holy thistle, Mary thistle; Latin names: *Silybum marianum* (L.) Gaertn, synonym *Carduus marianus* L.

Worldwide, liver diseases constitute a significant and prevalent factor in mortality rates, with cirrhosis, in particular, accounting for approximately 1.16 million fatalities each year, positioning it as the 11th leading cause of death. While hepatitis B continues to be a substantial concern in numerous Asian countries, the primary drivers of cirrhosis in Western industrialized nations are alcohol consumption and non‐alcoholic fatty liver disease (NAFLD) (Asrani et al., 2019). The utilization of natural compounds for therapeutic applications has garnered increased interest in recent times. One such example is the historical use of the herbal remedy, milk thistle, scientifically known as *Silybum marianum*, which boasts a well‐documented track record of safety. Silymarin, an active extract derived from milk thistle, has been the subject of extensive preclinical investigations, revealing notable antioxidant and hepatoprotective attributes (Gheybi et al., 2023) (Figure 1).

Since ancient times, liver disorders have been treated with silymarin, a flavonoid compound obtained from milk thistle seeds (Adetuyi et al., 2021). But the 20th century saw a rise in the use of silymarin as a supportive treatment for liver illnesses. When silymarin was first identified as a possible treatment for acute liver injury, researchers were examining its hepatoprotective qualities, namely its capacity to neutralize toxins from toxic mushrooms (Arman et al., 2022). Because of their vital roles in metabolism, detoxification, and nutrition storage, livers are vulnerable to toxins, infections, and metabolic diseases. Chronic inflammation or damage to liver cells can lead to liver illnesses, including cirrhosis, fatty liver disease, and hepatitis (Wadhwa et al., 2022). Antioxidant qualities, anti‐inflammatory actions, and encouragement of liver cell regeneration are some of silymarin's protective benefits. By eliminating oxidative stress and scavenging free radicals, antioxidants slow the development of liver disorders (Marmouzi et al., 2021). The frequent cause of liver illnesses such as cirrhosis is inflammation of the liver, which can be prevented or reduced by anti‐inflammatory treatments. The ability of silymarin to regenerate may aid in the healing process by repairing damaged liver tissue and restoring normal liver function (Younis et al., 2016).

Among the diverse silymarin formulations available, Legalon® shines as a prominent product in the pharmaceutical market. Legalon® is a well‐established medication with a rich historical lineage, incorporating the Eurosil 85® formulation. Its esteemed reputation is primarily attributed to its exceptional oral bioavailability, which ensures efficient absorption into the bloodstream while preserving its intended therapeutic potency (Cheemerla & Balakrishnan, 2021; Tajmohammadi et al., 2018). The pharmacokinetic properties of Legalon® have been the subject of extensive research, delving into the drug's absorption, distribution, metabolism, and elimination within the body. Concurrently, its pharmacodynamic characteristics, which elucidate how the drug interacts with the body to achieve the desired therapeutic effects, have also undergone comprehensive scrutiny. These well‐documented attributes affirm Legalon® as a dependable and efficacious treatment choice. The Legalon® formulation takes center stage in the analysis of a substantial body of clinical data presented in this review (Tajmohammadi et al., 2018).

The primary objectives of this narrative review encompass providing an in‐depth overview of the pharmacological features of silymarin extract and conducting a thorough examination of the substantiating evidence supporting its use as an adjunctive therapy for individuals grappling with liver‐related conditions. No human subjects or animals were used in the writing of this article because it is based on studies that have already been done.

## 2 THE BURDEN OF LIVER DISEASE WORLDWIDE

Liver illnesses are a significant global health burden, accounting for a portion of global morbidity and mortality. Chronic liver illnesses can lead to more serious conditions like cirrhosis and hepatocellular carcinoma. These diseases are frequently linked to lifestyle factors like obesity, alcohol usage, and viral infections. The financial toll that liver illnesses take on due to medical expenses and lost productivity highlights the critical need for efficient treatment solutions (Cheemerla & Balakrishnan, 2021).

## 3 PHARMACOLOGY OF SILYMARIN

### 3.1 Chemical composition

**FIGURE 2:**
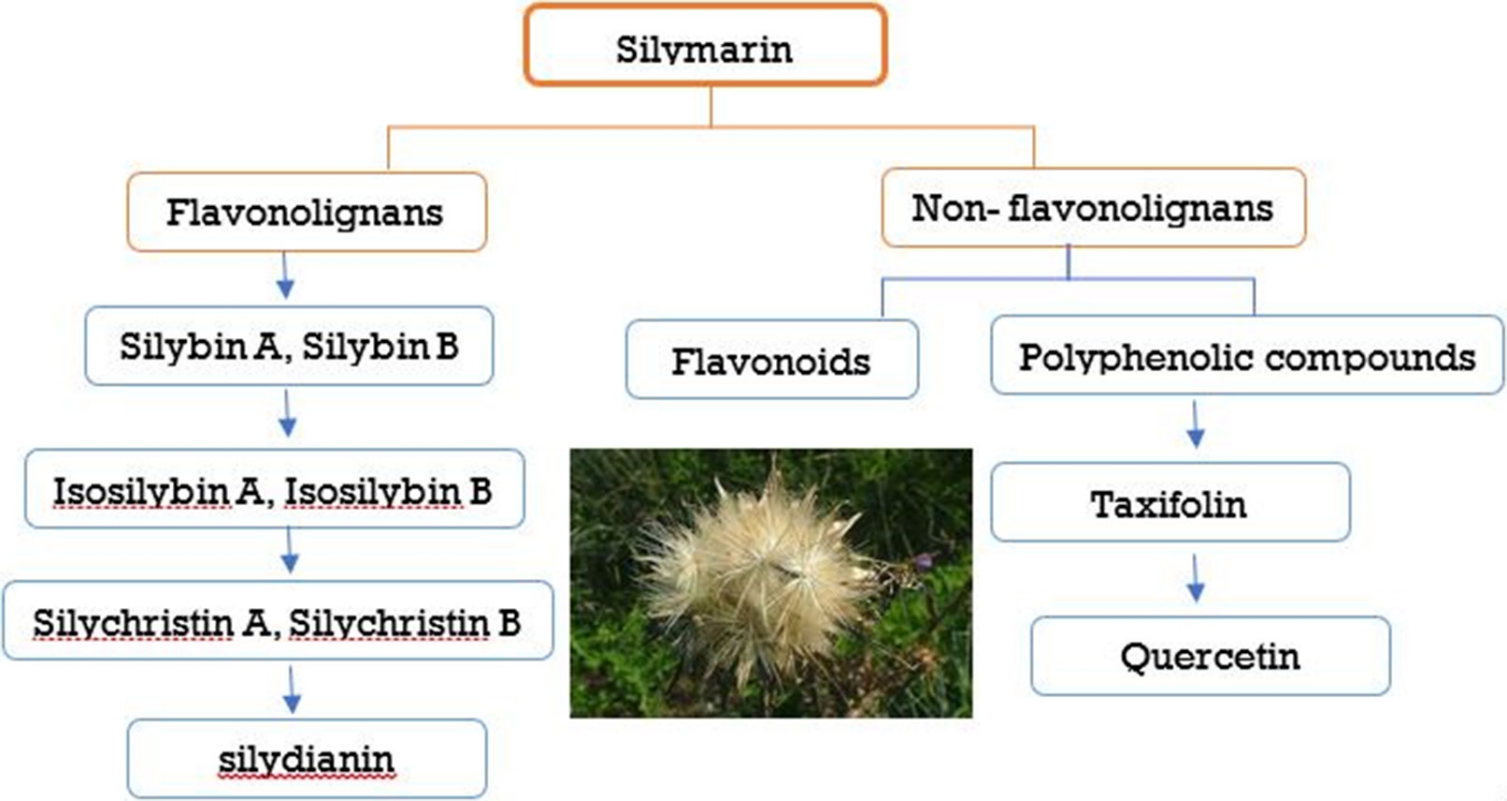
Different components of silymarin.

The herb *Silybum marianum*, sometimes referred to as milk thistle, has been used for therapeutic purposes for a very long time due to its ancient medicinal characteristics. Silymarin, which is taken from the seeds and fruits of milk thistle, is the principal bioactive ingredient produced from this plant. In the first century AD, Dioscorides, a well‐known Greek physician and botanist, made important advances in the knowledge of therapeutic plants in ancient Europe. Dioscorides used milk thistle, which he knew had therapeutic properties, as an early treatment for snakebites (Cheemerla & Balakrishnan, 2021; Moon et al., 2020). Thistle was used for centuries, and English herbalist Nicholas Culpeper suggested using it in the sixteenth century to cure a variety of conditions, such as jaundice and to help get rid of stones (Akhtar et al., 2023). The therapeutic benefits of milk thistle have been understood and passed down through the ages. The observations of Dioscorides and the advice of Culpeper provide light on the early acceptance of milk thistle's therapeutic advantages. These historical reports add to the expanding body of knowledge about the possible medical uses of milk thistle and silymarin, the plant's extract. Milk thistle contains a complex mixture of flavonolignans (Figure 1) called silymarin, which has antioxidant properties, scavenging free radicals and mitigating oxidative stress in liver cells. It also has anti‐inflammatory effects by modulating cytokine levels and inhibiting inflammatory pathways, contributing to its hepatoprotective potential. Its rapid absorption and distribution in the liver are also significant. The effectiveness of milk thistle seed extracts, or “tinctures,” in treating liver problems was later established in the 19th century by the German scientist Johannes Gottfried Rademacher (Akhtar et al., 2023; Valková et al., 2020). (Figure 2).

**FIGURE 3:**
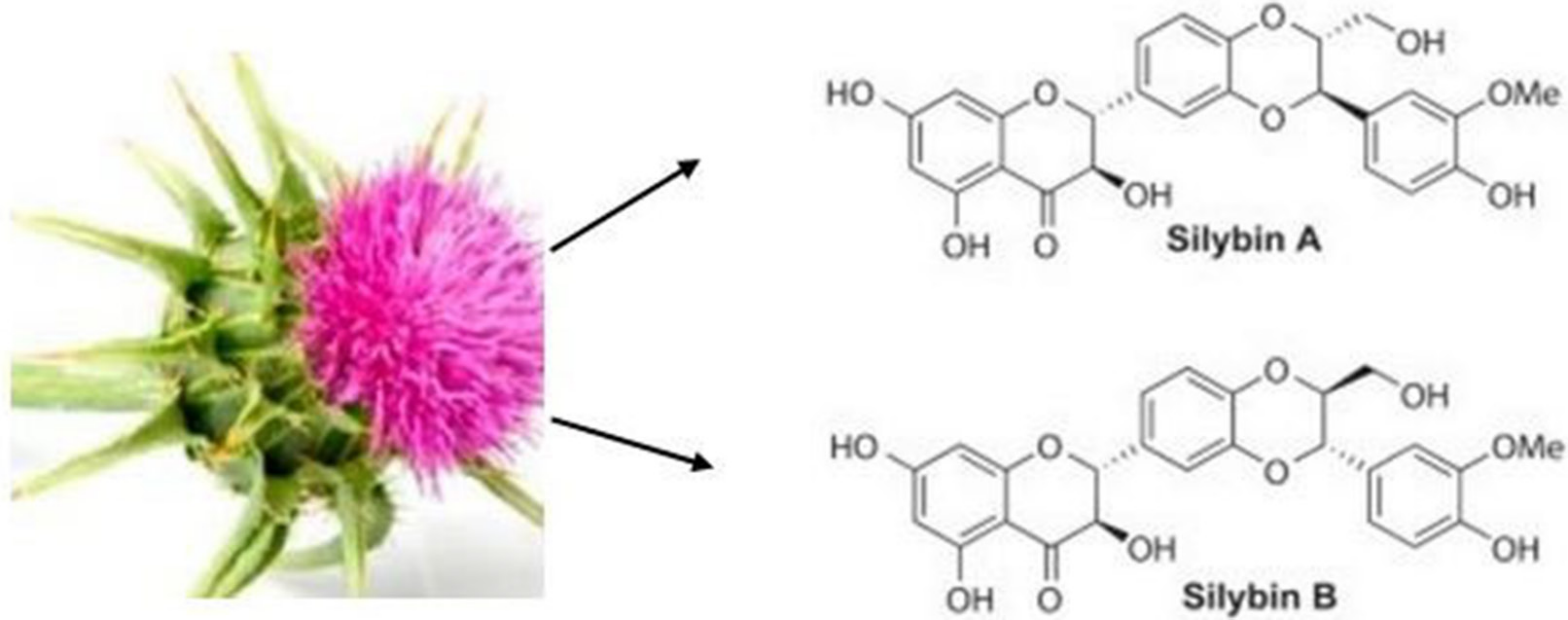
The two silibinin diastereoisomers, silibinin A and silibin B (C_25_H_21_O_10_), differ significantly in their chemical compositions.

These chemicals exhibit antioxidant capabilities in addition to several other biological functions (Alamgir & Alamgir, 2017; Gillessen & Schmidt, 2020). Silymarin contains the flavonolignan isomers silibinin, silibinin, silichristin, and silibinin as its primary constituents. The silibinin isomer of the silymarin complex, also known as silybin, is the most prevalent and physiologically active one. It makes up between 50 and 60% of the complex, with the remaining 35% made up of additional flavonolignan isomers such as silibinin, silibinin, and silichristin (Gillessen & Schmidt, 2020; Marceddu et al., 2022). Silibinin, a 482.44 g/mol polyphenolic flavonoid, reduces oxidative stress and promotes health due to its antioxidant properties. Further study explores potential medical disorders using silibinin (Křen & Valentová, 2022). With the molecular formula C25H22O10, silibinin contains the compounds silibinin A and silibinin B, which have distinct atom arrangements but similar molecular formulas (Figure 3). Advanced analytical techniques like HPLC and mass spectrometry have enabled precise quantification of silymarin components, enhancing our understanding of its pharmacological actions. Studies show variations in silymarin levels during milk thistle growth, emphasizing optimal harvesting times for therapeutic efficacy. Milk thistle's historical use validates modern research, highlighting its potential for therapeutic applications, especially in liver diseases (Surai, 2015). After consumption, silibinin is transformed by the liver using phases I and II of the biotransformation process. During phase II reactions, conjugation activities produce glucuronide and glucuronide sulfate derivatives (Musazadeh et al., 2022) (Figure 3).

### 3.2 Silymarin's historical development and commercial preparations

German researchers at the University of Munich made a breakthrough in isolation techniques that led to the discovery of silymarin in 1968, a pivotal moment in the history of the compound (Bhardwaj et al., 2023). A patent was subsequently sought for silymarin as a specialized treatment for liver issues by the German herbal medicine manufacturer Madaus after realizing its potential. The first commercial version of silymarin was created in part by the German business Rottapharm/Madaus, with its headquarters in Cologne. The European Pharmacopeia 01/2005's analytical conformity standards are met by this formulation, in particular, the section on “Milk Thistle fruit.” Silymarin has received approval from numerous countries to treat liver problems in Asia, America, Europe, Africa, and Australia (Gillessen & Schmidt, 2020). It comes in a variety of forms, including pills and capsules with varied silymarin concentrations. Depending on the individual commercial formulation utilized, the suggested daily dosage normally ranges from 420 to 600 mg. Typically, a dose of 140 mg administered three times per day has been employed in the majority of clinical investigations (Gillessen & Schmidt, 2020).

### 3.3 Pharmacokinetics

Indeed, the lipophilic structure and restricted water solubility of silymarin, the active compound produced from milk thistle, affect its bioavailability. About 20% to 50% of the silymarin crude extract that is consumed is absorbed by the digestive tract. Silymarin, a flavonolignan, is a drug with pharmacological effects due to its lipophilic nature. It dissolves in lipid‐rich environments, facilitating absorption in the digestive tract. However, its limited water solubility challenges its absorption, as water is the primary medium for nutrient absorption. Factors like formulation, dietary components, and metabolism can influence bioavailability. To improve bioavailability, formulations have been developed using techniques like micronization and phospholipid complexation. Understanding silymarin's bioavailability is crucial for optimizing its therapeutic efficacy, and research is ongoing to explore innovative approaches (Mihailović et al., 2023; Valentová et al., 2020). Formulation experts have created unique techniques to increase silymarin formulations' oral bioavailability and solubility. The active ingredient, silibinin, is present in varying amounts, solubilities, and oral bioavailabilities in commercially available silymarin preparations (Zhu et al., 2013).

Rottapharm and Madaus invented co‐precipitation processing in 1995, which significantly improved the production of silymarin. This method produced high‐quality silymarin with improved dissolving properties, achieving purities ranging from 90% to 96% and high silibinin contents of approximately 60%. This process enhances the purity and concentration of key active components, particularly silibinin, which is a major bioactive constituent responsible for therapeutic effects. In 2014, a patent was granted, highlighting the uniqueness and proprietary nature of this processing method. The patent encourages further research and investment in refining and applying this method for silymarin‐based products, highlighting the ongoing efforts in the pharmaceutical and nutraceutical industries (Gillessen & Schmidt, 2020) under the trade name Eurosil 85®. A common treatment for silymarin, a medication with a high concentration of silibinin and a high rate of biodissolution, is Eurosil 85®, a standardized pharmaceutical product. When taken daily, it provides 420 milligrams of silymarin, equivalent to 250 milligrams of silibinin. This high concentration of silibinin makes Eurosil 85® a reliable and consistent form of silymarin for clinical research and therapeutic applications. The product's specific dosage and composition ensure accurate assessments of its efficacy and safety in various clinical settings. Eurosil 85® has significantly contributed to our understanding of silymarin's therapeutic potential through rigorous scientific investigation (Abenavoli et al., 2011; Di Costanzo & Angelico, 2019).

**FIGURE 4:**
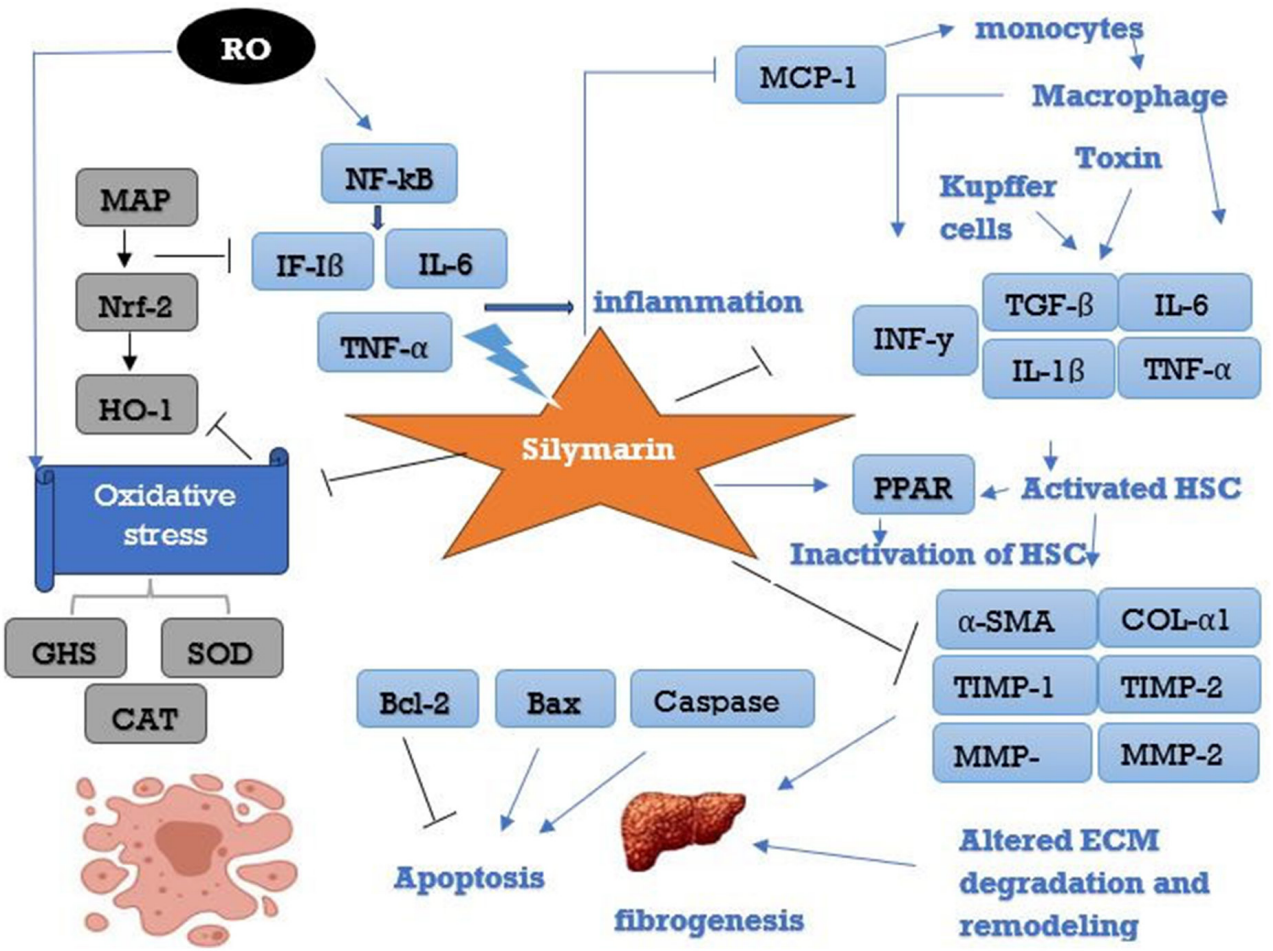
Different hepatoprotective modes of action of silymarin. CAT, catalase; COL‐α1, collagen α1; IL‐1β, interleukin‐1 beta; IL‐6, interleukin‐6; INF‐γ, interferon‐gamma; MAPK, mitogen‐activated protein kinase; MCP‐1, monocyte chemoattractant protein‐1; MMPs, matrix metalloproteinases; NF‐κB, nuclear factor kappaB; Nrf‐2, nuclear factor erythroid 2‐related factor 2; PPARα, peroxisome proliferator‐activated receptor alpha; ROS, reactive oxygen species; SOD, Superoxide dismutase; TGF‐β, transforming growth factor‐beta; TIMP 1,2, Tissue inhibitor of metalloproteinases 1,2; TNF‐α, tumor necrosis factor‐alpha; α‐SMA, α‐smooth muscle actin.

The special combination of silymarin is easily absorbed when taken orally. The main ingredient in silymarin, silibinin, normally takes two to four hours to reach its peak plasma concentration after oral consumption, and it has a 6‐hour plasma half‐life (Hawke et al., 2010; Zhu et al., 2013). Phase I biotransformation involves reactions like oxidation, reduction, and hydrolysis to make a compound more reactive. This is followed by phase II conjugation reactions, where the compound is conjugated with endogenous molecules like glucuronic acid, sulfate, or amino acids. This makes the compound more water‐soluble and facilitates its excretion through urine or bile. Understanding these processes is crucial for determining the pharmacokinetics of silymarin and its components, providing insights into its potential therapeutic benefits. As silibinin and the other components of silymarin are absorbed into the liver cells via the gastrointestinal (GI) tract, they both go through important phase I and phase II biotransformation processes (Tighe et al., 2020). These biotransformation activities are essential to the metabolism of xenobiotics, which include naturally occurring substances like silibinin, in order to aid in the body's removal from them (Figure 4).

One essential component of silymarin, silibinin, is metabolized and eliminated in large part by the enterohepatic circulation. Eighty percent of the silibinin is eliminated as conjugates of glucuronide and sulfate via the bile. Through this process, silibinin is changed into forms that are more easily excreted, like glucuronide and sulfate conjugates, which are soluble in water. It is estimated that between 20% and 40% of the bile containing silibinin conjugates is reabsorbed in the enterohepatic circulation. After this reabsorption takes place in the small intestine, the portal circulation returns the recycled silibinin to the liver. This recycling process extends silibinin's duration of action by keeping it in the systemic circulation longer. Feces remove residual silibinin from the body through complex interactions between enterohepatic circulation, excretion mechanisms, and hepatic metabolism. This dual pathway of elimination, through bile and feces, affects the pharmacokinetics of silibinin and maintains its presence. Understanding the body's use and recycling of silibinin, its bioavailability, and persistence in the systemic circulation is crucial for comprehending its overall bioavailability and persistence (Abenavoli et al., 2018; Teschke, 2018).

Studies investigating drug interactions and their effects on the cytochrome P450 (CYP450) enzymes have all used human primary hepatocytes, Caco‐2 cells, and liver microsomes. Silymarin demonstrated modest inhibition of some CYP450 enzymes at a supratherapeutic dose and slight to moderate inhibition of others. Since silymarin's therapeutic concentration is substantially lower than that of silibinin, silymarin is unlikely to cause hepatic medication interactions when taken as prescribed (Bijak, 2017). There are no significant effects of milk thistle on various CYP450 enzymes, according to clinical studies and trials involving healthy volunteers (Tvrdý et al., 2021).

Even though silymarin has been shown to help diabetics with their blood sugar levels, the danger of hypoglycemia, when taken with antidiabetic medications, is still merely a theory because there are no recorded instances of clinical data to support this additive effect. Other possible medication interactions include disruption of estrogen therapy, slowed glucuronidated drug clearance, and accelerated absorption of P‐glycoprotein substrates. Furthermore, silymarin and silibinin may interact with statins, preventing their movement into and out of the liver. Silymarin should be used with caution when combined with CYP450 enzyme substrates or particular drugs since more research is needed to fully understand the significance of these potential interactions. Silymarin did not affect the rosuvastatin pharmacokinetics in a study with healthy males, while sirolimus' apparent clearance was reduced in a trial with hepatically compromised renal transplant patients (Tvrdý et al., 2021).

### 3.4 Pharmacodynamics

Silymarin has been used in traditional medicine for a very long time, and its possible hepatoprotective benefits have been well investigated. Silymarin, which is made up of flavonolignans such as silybin, silydianin, and silychristin, has anti‐inflammatory, antifibrotic, and antioxidant qualities. Current studies have examined the molecular processes that underlie silymarin's effects, as well as how it affects inflammation, oxidative stress, and other signaling pathways connected to liver disorders.

### 3.5 Antioxidants properties

Reactive oxygen species (ROS), which are mostly linked to detoxification activities, are spontaneously created in the liver as part of necessary metabolic reactions. However, excessive free fatty acid oxidation or exposure to high quantities of toxins, such as alcohol or hepatotoxic medications, can result in aberrant ROS generation and the depletion of endogenous antioxidants. In a variety of cell types, such as hepatocytes, Kupffer cells, endothelial cells, and inflammatory leukocytes, ethanol is known to encourage the generation of free radicals (Rombolà et al., 2020). This imbalance also referred to as “oxidative stress,” has been linked to the onset of several liver conditions, including liver fibrosis (Sprouse & Van Breemen, 2016).

In vitro, research has demonstrated that silibinin has potent anti‐ROS qualities, including the capacity to scavenge peroxyl anions and hydroxyl, superoxide anion radicals, hypochlorous acid, and nitric oxide. It has been observed in several model systems, including rat liver microsomes, leukocytes, fibroblasts, erythrocytes, endothelial cells, and human platelets (Pellicoro et al., 2014). It has been demonstrated that silibinin treatment reduces ROS production in Kupffer cells (with a 50% inhibition concentration of 80 mol/L) (Conde de la Rosa et al., 2022). By making more of the enzyme's building block (cysteine) available for biosynthesis, silymarin, the precursor to silibinin, can increase glutathione production in the liver and hence increase the liver tissues' antioxidant capacity (Sozen et al., 2015).

In several different methods, silymarin protects liver cells. First off, it prevents lipid peroxidation, which supports the maintenance of endogenous protective antioxidant glutathione levels and stabilizes membrane permeability (Wadhwa et al., 2022). Silymarin, a natural flavonoid complex from milk thistle, has been shown to inhibit the production of pro‐inflammatory cytokines, such as TNF‐α, IFN‐γ, IL‐2, and IL‐4, which are crucial in the inflammatory cascade and immune response. This has significant therapeutic implications for hepatoprotection, as it shields against damage from harmful substances like carbon tetrachloride, which triggers oxidative stress and inflammation. Silymarin's mechanism involves suppressing NF‐κB activation, which regulates the expression of pro‐inflammatory genes. This makes silymarin a promising candidate for addressing hepatic disorders linked to inflammation and oxidative stress (Camini & Costa, 2020; Perez‐Araluce et al., 2024).

The regulation of inflammation in the liver is achieved by inhibiting hepatic nuclear factor kappa B (NF‐κB) activation. NF‐κB is a key transcription factor involved in inflammation, immune response, and cell survival. It downregulates inflammatory mediators like interleukins, TNF‐α, and iNOS, which are involved in various diseases. NF‐κB inhibition also modulates cellular signaling pathways, such as the mitogen‐activated protein kinase (MAPK) pathway. It may also impact oxidative stress pathways, potentially mitigating damage to cellular components. Therefore, targeting NF‐κB as a therapeutic strategy is crucial in hepatic inflammatory conditions (Perez‐Araluce et al., 2024; Taleb et al., 2018).

Silymarin, a natural compound from the milk thistle plant, has a protective mechanism that prevents the reception of xenobiotics, including toxic substances found in mushrooms, by hepatocyte surfaces. It inhibits organic ion uptake transporters, which facilitate the transport of substances across cell membranes. This prevents harmful substances from entering hepatocytes, reducing the risk of cellular damage. Silymarin's interference with these transporters is part of its broader hepatoprotective mechanism, contributing to the overall defense against toxic insults. Its inhibition of transporters, including OATPs and OCTs, may also affect members of the solute carrier family. This makes silymarin a promising natural compound for safeguarding the liver against environmental poisons (Gharagozloo et al., 2010). Its ability to inhibit the production of tumor necrosis factor‐alpha (TNF‐α), a cytokine linked to inflammatory reactions that is frequently brought on by toxic substances like the amanitin toxin present in lethal mushrooms, is especially remarkable (Mansour et al., 2022). The protective actions of silymarin against TNF‐α and other inflammatory mediators are intricately linked to its antioxidant activities. Silibinin, a major active constituent of silymarin, is renowned for its potent antioxidant effects, which involve scavenging reactive oxygen species (ROS) and inhibiting oxidative stress pathways (Kadoglou et al., 2022). These antioxidant properties are frequently cited as the primary drivers of silibinin's hepatoprotective benefits.

Toxin exposure can cause oxidative stress in hepatocytes, leading to the generation of reactive oxygen species (ROS) and oxidative stress. Silimarin, specifically silibinin, counteracts these harmful effects by preventing TNF‐α development and exhibiting antioxidant activities. This not only mitigates inflammatory responses but also addresses oxidative damage that contributes to liver injury. Silimarin also modulates various signaling pathways, including the NF‐κB pathway, further contributing to its anti‐inflammatory and antioxidant effects. This makes silymarin a promising natural compound for protecting the liver against environmental poisons and potentially treating liver damage caused by toxic substances.

### 3.6 The ability to reduce inflammation

Hepatic fibrosis that progresses and cirrhosis formation are both closely linked to chronic inflammation (Basu et al., 2023). In the beginning and development of hepatic inflammation in a variety of liver illnesses, oxidative stress is thought to represent a common underlying mechanism (Surai, 2015). The key transcriptional regulator of the inflammatory response NF‐B controls the signaling pathways that cause inflammation in the liver (Goh et al., 2020). The majority of chronic liver diseases, such as viral hepatitis, non‐alcoholic and alcoholic fatty liver disease, and biliary liver disease, are also accompanied by NF‐B activation (Chupradit et al., 2022; Kondylis et al., 2017; Tanwar et al., 2020; Younossi et al., 2016; Zhou et al., 2018). Silymarin may have anti‐inflammatory properties that limit the production of inflammatory mediators like NF‐B and inflammatory metabolites like prostaglandin E2 (PGE2) and leukotriene B4 (LTB4), according to growing evidence (Liu et al., 2023).

Innate immunological responses and host defense are aided by Kupffer cells, the resident liver macrophages, which produce and secrete inflammatory mediators (Rani et al., 2024). Even at low doses (15 mol/L), silymarin showed poor suppression of LTB4 but substantial inhibition of PGE2 synthesis in isolated rat Kupffer cells. A contributing component to silymarin's anti‐inflammatory activities may be the specific reduction of LTB4 production by Kupffer cells and maybe other cell types (Křen & Valentová, 2022). Silymarin's anti‐inflammatory properties are attributed to its ability to modulate inflammatory mediators in Kupffer cells. Its significant inhibition of PGE2 synthesis aligns with its anti‐inflammatory properties, as PGE2 is known for its pro‐inflammatory actions. However, the less pronounced suppression of LTB4 suggests a nuanced response, suggesting that silymarin's impact on specific inflammatory pathways may vary. The specific reduction of LTB4 production by Kupffer cells, attributed to silymarin, is a notable aspect of its anti‐inflammatory activities. This regulation of LTB4 by silymarin implies a targeted intervention in the inflammatory cascade. The complex interplay between silymarin and Kupffer cells adds complexity to understanding its immunomodulatory effects in the context of hepatic inflammation (Gharagozloo et al., 2010).

### 3.7 Effects on fibrosis

In vitro and animal models using silibinin have demonstrated antifibrogenic effects (Liu et al., 2023; Reddy et al., 2017). Hepatic stellate cells (HSCs), a kind of pericyte unique to the liver, are activated as a result of chronic liver tissue injury in hepatic fibrogenesis. Myofibroblasts, which are produced when HSCs are activated, deposit collagen fibers and cause liver cirrhosis (Mansour et al., 2022). Silymarin's anti‐fibrogenic action has to be proven in an animal model of hepatic fibrosis in non‐human archbishops following ongoing alcohol therapy (Liu et al., 2023). Alcohol consumption (50% of daily calories) and a nutritionally sound diet were given to baboons for three years, and hepatic biopsy samples from these animals revealed increased collagen type I. However, silymarin therapy given concurrently with alcohol consumption significantly decreased the upsurge in hepatic collagen type I caused by alcohol, as demonstrated in the study (Liu et al., 2023).

### 3.8 Changes in insulin resistance

Non‐alcoholic fatty liver disease (NAFLD) is closely linked to insulin resistance, and silibinin, a major component of silymarin, has been shown to have promising effects in mitigating insulin resistance in a rat model of NAFLD. Silibinin's positive outcomes in a rat model include reducing visceral obesity, improving lipolysis, and suppressing gluconeogenesis, which is associated with insulin resistance and elevated blood glucose levels. These findings suggest that silibinin may modulate adipose tissue, a major contributor to insulin resistance, and alleviate the burden of lipid accumulation in the liver. The multifaceted actions of silibinin on visceral obesity, lipolysis, and gluconeogenesis in the rat model provide a promising foundation for further exploration of its potential as a treatment strategy for NAFLD and insulin resistance in humans (Chen et al., 2017; Li & Zeng, 2020). These results demonstrate the silibinin's potential to modulate insulin resistance and address the underlying causes of NAFLD.

Silymarin and its bioactive components exhibit multifaceted anti‐cancer properties through various mechanisms. They suppress cellular growth and proliferation by modulating the MAPK signaling pathway, inducing apoptosis in numerous cell lines. Silymarin influences cell cycle progression, inhibiting overexpressed regulators of the cell cycle, such as cyclins, CDKs, and CDKIs. It also perturbs several signaling pathways, including the Notch pathway, PI3K/Akt, and Wnt/β‐catenin, to impede cancer progression and invasion. Silymarin significantly inhibits matrix metalloproteinases (MMPs), essential for cancer metastasis, and enhances mitochondrial fusion while reducing inflammation and chemokine receptor expression, ultimately curbing tumor growth, proliferation, and metastasis. Additionally, silymarin down‐regulates VEGF expression, contributing to anti‐angiogenic effects, and has the potential to reverse STAT‐3‐associated cancer drug resistance. These findings underscore the versatile and promising role of silymarin in combating cancer (Fallah et al., 2021; Koushki et al., 2023; Robinson & Mann, 2010).

## 4 SILYMARIN'S IMPACTS

### 4.1 Cirrhosis and alcohol‐related liver disease

The accumulation of excessive fat in the liver is the defining feature of the condition known as fatty liver disease (FLD), which can result in more severe liver disorders. Alcoholic fatty liver disease (AFLD) is the early stage of alcohol‐related liver damage in those who consume excessive amounts of alcohol (Kim et al., 2012; Wenfeng et al., 2014; Yao et al., 2013). Silymarin has been the subject of numerous clinical trials involving individuals with liver cirrhosis and/or liver disease brought on by alcohol (Table 1) (Amini et al., 2023; European Association for the Study of the Liver [EASL], 2018; Frank et al., 2021; Hussain et al., 2022; Meng et al., 2022; Mirhashemi et al., 2022; O'Shea et al., 2010; Saller et al., 2001; Solhi et al., 2014; Thuy et al., 2015; Zarif‐Yeganeh & Rastegarpanah, 2019). Patients with liver cirrhosis, primarily caused by alcohol, were the focus of six of these investigations (EASL, 2018; Kim et al., 2012; Wenfeng et al., 2014; Yao et al., 2013). Four of these trials examined the effect of silymarin on clinical outcomes, including death. In particular, the trial (Koushki et al., 2023), which involved giving silymarin (a formulation derived from Eurosil 85®) to patients with cirrhosis, had a significant influence on mortality. The study involved 170 patients in all, and the median length of observation was 41 months. These were divided into 87 individuals who got silymarin (420 mg/day), and 83 patients who received a placebo. Compared to the placebo group, the silymarin group had fewer liver‐related fatalities. In subgroup analyses, patients with cirrhosis due to alcoholism and those with less severe cirrhosis had lower mortality rates (Koushki et al., 2023; Younis et al., 2016). Similar to this, randomized controlled trial looked at the survival rates of patients who received silymarin or a placebo over two years. Although there was no statistically significant difference in overall mortality, an examination of the silymarin group's hepatitis C patient subgroup showed a clear trend toward fewer fatalities (Wenfeng et al., 2014).

**TABLE 1:** Clinical trials are underway to evaluate the effectiveness of silymarin in treating liver diseases such as cirrhosis, alcoholic liver disease, and non‐alcoholic steatohepatitis or NAFLD.

Treatments	Outcome with silymarin	Duration	Number of subjects	Conditions	References
Silymarin 420 mg/day	An improvement in liver function markers, Alanine transaminase, Aspartate aminotransferase, and Histopathology	4 weeks	97	Liver disease	European Association for the Study of the Liver (EASL, 2018)
Silymarin 420 mg/day	No significant effect	3 months	116	Alcoholic liver disease (cirrhosis)	O'Shea et al. (2010)
Silymarin 420 mg/day	The 4‐year survival rate has improved, especially in ALD patients with cirrhosis and low‐severity diseases (Child class A).	41 months	170	Alcoholic liver disease or NAFLD (cirrhosis)	Mirhashemi et al. (2022)
Silymarin	Synthesis of bilirubin, procollagen, aspartate aminotransferase, and alanine transaminase has improved.	6 months	36	Alcoholic liver disease	Meng et al. (2022)
Silymarin 420 mg/day	Enhancing anti‐oxidative systems (↓ MDA, ↑ glutathione)	6 months	NA	Alcoholic liver disease	Zarif‐Yeganeh and Rastegarpanah (2019)
Silymarin 210 mg/day	Liver function, mortality, or clinical course are unaffected.	15 months	59	Alcoholic liver disease (cirrhosis)	Solhi et al. (2014)
Silymarin 600 mg/day + standard treatment	A reduction in the daily insulin demand, an improvement in blood sugar (including fasting), glycosylated hemoglobin, MDA, ↓ alanine transaminase, and aspartate aminotransferase	12 months	60	Insulin‐treated type II diabetes mellitus with alcoholic cirrhosis	Frank et al. (2021)
Silymarin 450 mg/day	No impact on the survival rate or development of liver disease	2 years	200	Alcoholic liver disease with cirrhosis	Gillessen and Schmidt (2020)
Silymarin 450 mg/day	↓ MDA and the procollagen type III aminoterminal propeptide	6 months	49	Alcoholic liver disease with cirrhosis	Amini et al. (2023)
Silymarin 420 mg/day	Transaminases and lipid markers are decreased, and subjective well‐being is improved	2 months	70	Non‐alcoholic steatohepatitis (50) or Non‐alcoholic fatty liver disease (20)	Thuy et al. (2015)
Silymarin 280–420 mg/day	Liver function indicators and overall quality of life are improving.	4 months	190	NAFLD	Perumpail et al. (2017)
Silymarin 420 mg/day + vitamin E	Improvements in liver size, indices, and biometric measurements including abdominal circumference and body mass index	90 days	78	Non‐alcoholic fatty liver disease and metabolic syndrome	Sonmez et al. (2021)
Silymarin 2100 mg/day	The parameters of noninvasive fibrosis indicators, liver function, and liver histology have all improved	48 weeks	99	Non‐alcoholic steatohepatitis	Lee et al. (2022)

A meta‐analysis of clinical data found that, overall, silymarin had a 0.53 odds ratio for liver‐related mortality compared to placebo across all included studies, which is a 47% risk reduction. Patients receiving silymarin showed a lower overall death rate from liver illness compared to those getting a placebo. Users of silymarin had a decreased percentage of hospital admissions for liver‐related problems, according to another study (EASL, 2018). However, it should be noted that some of the trials that examined mortality as a result could not have had sufficient power or time to establish a significant influence on survival. A Cochrane review (Meng et al., 2022; Saller et al., 2001) discovered that milk thistle silymarin, compared to a placebo or no treatment, significantly reduced liver‐related mortality. When only high‐quality trials were considered, this advantage was not, however, consistently observed. It will take more extensive research to properly comprehend how silymarin influences mortality from liver‐related causes.

It was repeatedly observed that silymarin‐treated participants had lower ALT and AST levels than those who received a placebo (Mirhashemi et al., 2022; O'Shea et al., 2010). For instance, silymarin therapy dramatically improved liver functions as seen by a drop in AST and ALT levels in a randomized study involving persons with acute, moderate, and subacute hepatic disease caused by alcohol intake (O'Shea et al., 2010). Indicators of oxidative stress have improved, according to much research (Hussain et al., 2022). Patients with chronic alcoholic liver disease who underwent silymarin treatment saw a significant improvement for six months in their antioxidant defense, placebo‐controlled study. This improvement was shown by increased glutathione peroxidase, free‐SH group, and superoxide dismutase activity levels in the serum. In another trial, silymarin was used for the long‐term treatment of patients with alcoholic cirrhosis, insulin‐treated diabetes, lipoperoxidation, and insulin resistance. There were signs of this in the form of reduced levels of glycosylated hemoglobin and systemic inflammatory symptoms (Yao et al., 2013).

However, it should be noted that some of the trials that examined mortality as a result could not have had sufficient power or time to establish a significant influence on survival. A Cochrane review discovered that milk thistle silymarin, compared to a placebo or no treatment, significantly reduced liver‐related mortality. When only high‐quality trials were considered, this advantage was not, however, consistently observed. It will take more extensive research to properly comprehend how silymarin influences mortality from liver‐related causes (Table 1).

### 4.2 NAFLD with non‐alcohol steatohepatitis

NAFLD is a common chronic liver disorder that is not brought on by drinking excessive amounts of alcohol, along with multiple disorders like central obesity, insulin resistance, diabetes type II, and dyslipidemia (Solhi et al., 2014). It now accounts for up to 33% of liver disease cases in Western nations and has become a major worldwide health concern. As obesity and related illnesses are becoming increasingly common, NAFLD is spreading throughout Eastern nations (Frank et al., 2021). The term “NAFLD” is used to describe a wide range of conditions, including simple steatosis (benign fat accumulation), end‐stage liver disease, progressive fibrosis, cirrhosis, and hepatic inflammation (steatohepatitis) (Perumpail et al., 2017; Younossi et al., 2019). About 20% of patients who have NAFLD also develop NASH, a more severe form of the disease (Meng et al., 2022; Solhi et al., 2014; Thuy et al., 2015; Zarif‐Yeganeh & Rastegarpanah, 2019).

Non‐alcoholic fatty liver disease (NAFLD) therapy primarily involves dietary modifications and increased physical exercise, but many patients struggle to maintain these changes. Oxidative stress is a key factor in the progression from basic steatosis to non‐alcoholic steatohepatitis (NASH), contributing to the disease's pathogenesis. Understanding the interplay between oxidative stress and NAFLD stages is crucial for developing targeted interventions (Lee et al., 2022; Sonmez et al., 2021). Oxidative stress triggers inflammation and cellular damage in the liver, leading to the progression from simple steatosis to the more severe NASH phenotype. Research has identified molecular players like Nrf2 and antioxidant enzymes that can mitigate oxidative stress within hepatocytes. Understanding these molecular mechanisms can provide a scientific foundation for exploring innovative therapeutic approaches, offering hope for more effective and sustainable strategies in mitigating NAFLD progression, especially in cases where conventional lifestyle changes are challenging (Drescher et al., 2019; Tarantino et al., 2019; Younossi & Henry, 2022). In addition to preventing or postponing oxidative stress and hepatocyte damage, endogenous antioxidants also operate as reactive oxygen species (ROS) scavengers. The most common cellular antioxidant is glutathione which protects from the harmful effects of ROS by shielding hepatocytes. Recent research has shown that using free radical scavengers and antioxidant medications can improve the biochemical and histopathological parameters in NASH (García‐Ruiz & Fernández‐Checa, 2018; Pisoschi et al., 2021; Shrestha & Pradhananga, 2022).

Vitamin E has been explored in clinical trials for treating non‐alcoholic steatohepatitis (NASH) and non‐alcoholic fatty liver disease (NAFLD). A large randomized trial showed significant improvement in adult NASH patients receiving vitamin E therapy at a dosage of 533.6 mg/day for 96 weeks. This improvement likely relates to vitamin E's antioxidant properties, which neutralize reactive oxygen species and protect cells from oxidative stress. However, the efficacy and safety of vitamin E therapy for NASH/NAFLD treatment remain debated, and concerns about potential adverse effects and long‐term consequences of high‐dose vitamin E supplementation remain. The mixed results highlight the complexity of these conditions and suggest that treatment approaches may need to be tailored to individual patients. Decisions should be made in consultation with healthcare professionals based on a comprehensive evaluation of an individual's health status (García‐Ruiz & Fernández‐Checa, 2018; Pisoschi et al., 2021).

Currently, patients with biopsy‐proven NASH who do not have diabetes should simply get vitamin E therapy. Both NAFLD and NASH have been studied as potential targets for silymarin therapy in the future (Handayani & Prijanti, 2024; Pisoschi et al., 2021; Podszun et al., 2020), especially the formulation made from Eurosil 85®. In pilot research, patients with NAFLD or NASH received either a diet‐only intervention or silymarin supplementation for two months. The impact of the therapy was evaluated by the results. Silymarin enhanced hepatic ultrasonography parameters and decreased or normalized transaminase levels, which are markers of liver function, according to the results (Curcio et al., 2020; Sodum et al., 2021).

In different research, NAFLD patients received either a diet‐only intervention or a brand‐new silymarin with vitamin E dietary supplement for three months. As outcome markers, the established measurements of liver steatosis known as the hepatic steatosis index and the lipid accumulation product were used. Both indices and biometric assessments demonstrated that the silymarin or vitamin E group did significantly better than the placebo group. These results imply that the use of silymarin or vitamin E as a nutritional supplement in persons with simple NAFLD, for whom diet and exercise are the advised course of treatment, maybe more beneficial in improving outcomes and encouraging long‐term adherence to lifestyle changes (Curcio et al., 2020; Handayani & Prijanti, 2024).

Only a slight improvement in NASH patients' conditions was seen in early studies using the commercial silymarin formulation (Eurosil 85®) at the recommended dosage of 420 mg/day. To further investigate the possible benefits, clinical research with a larger dose of silymarin (up to 2100 mg/day) was carried out over 48 weeks (de Avelar et al., 2017) with individuals who had biopsy‐confirmed NASH. Even though the primary objective, a 30% improvement in the NAFLD as well as NASH from baseline Activity Score on liver biopsy, did not demonstrate a statistically significant difference, more patients in the silymarin group displayed detectable improvements in fibrosis compared to the placebo group. The NAFLD fibrosis score, the fibrosis‐4 score, and the AST to platelet ratio index all exhibited improvements in the silymarin group, while the placebo group did not show any improvement in their results (Sodum et al., 2021).

### 4.3 Liver failure resulting from amotoxin

The severity of hyperacute liver failure brought on by consuming mushrooms that contain amatoxin depends on how much was taken in. RNA polymerase II, a crucial enzyme for the function of hepatocytes, is severely inhibited by amatoxin. This makes it a popular model for toxic‐caused liver failure. Despite the difficulties in carrying out prospective studies specifically investigating the use of silymarin for this condition brought on by mushroom poisoning, the administration of a parenterally administered silibinin‐based formulation is the preferred treatment approach in cases of amatoxin‐induced liver failure (Fu et al., 2022). For amatoxin‐ induced liver failure to be successfully managed, early identification and fast intravenous medication initiation are essential.

### 4.4 Drug‐related liver damage

Drug‐induced liver damage is a serious issue since numerous drugs can result in hepatotoxicity through either their active metabolites or direct hepatic metabolism, which raises the risk of morbidity and mortality. Anti‐tuberculosis medications (ATDs) are an example since they may call for modifying the course of treatment or adjusting the dosage (Chalasani et al., 2018; Sorrentino et al., 2015). Previous studies looked into how herbal medicines, phytochemicals, and nutritional supplements might be used to stop or lessen the hepatotoxic effects of certain pharmaceuticals (French et al., 2011). Silymarin's potential to stop DILI brought on by ATDs has been well‐researched. ATDs with silibinin capsules (70 mg, three times daily) or ATDs alone in a multicenter trial involving 565 patients did not significantly differ in terms of liver injury, DILI diagnosis, or discontinuing ATD therapy due to liver injury and associated symptoms after eight weeks of therapy. But the incidence of anorexia and nausea was lower in the silibinin group, indicating less liver damage (Mengs et al., 2012).

However, silymarin dramatically decreased the incidence of ATD DILI (32.1%) compared to placebo (3.7%) after four weeks of treatment, according to a smaller trial involving 55 patients. This study also showed that silymarin retained superoxide dismutase, an antioxidant enzyme linked to a lower risk of liver damage (Tao et al., 2019). The preventative effects of silymarin against ATD DILI were further confirmed by a recent meta‐analysis of five randomized controlled studies with a combined total of 1198 participants. The research revealed improved liver function as seen by lower levels of ALT, AST, and alkaline phosphatase, as well as a considerably lower likelihood of developing DILI four weeks after starting silymarin treatment (Sorrentino et al., 2015).

A real‐world observational trial involving 190 patients with probable drug‐induced liver injury (DILI) found that the commercial silymarin formulation, Eurosil 85®, significantly reduced hepatotoxicity signs and symptoms. The study also showed that silymarin improved overall quality of life for the patients, with the effects becoming more pronounced after two months of consistent therapy. The reduction in liver enzyme levels among the treated patients was a key metric, indicating the amelioration of hepatic stress and improved liver function. The trial's findings suggest silymarin's potential as a valuable adjunctive therapy for individuals experiencing DILI, fostering sustained improvements in liver health and overall well‐being over an extended treatment duration. Further research on silymarin's impact on specific DILI subtypes and potential synergies with conventional treatments may further enhance its clinical relevance (Bernal & Wendon, 2013).

Before treatment, a comprehensive evaluation of liver function is essential given the link between chemotherapeutic medications and DILI. In a study with 70 Chinese patients (*n* = 70) with acute lymphoblastic or acute myeloid leukemia who were receiving chemotherapy, those receiving silymarin (420 mg/day) in combination with diammonium glycyrrhizinate had a higher percentage of patients reporting no or mild DILI than those receiving only diammonium glycyrrhizinate (Baskaran & Sabina, 2017; Gu et al., 2015). Additionally, two pilot studies involving young patients with acute lymphoblastic leukemia assessed the treatment and preventative effects of formulations based on silymarin on chemotherapy‐induced liver damage. Significant improvements in liver enzyme profiles were seen, indicating that silymarin may be a secure and useful supportive care drug for chemotherapy patients (Gillessen et al., 2014; Luangchosiri et al., 2015). These results show the potential of silymarin as a supportive medicine to stop or lessen drug‐induced liver damage in a variety of therapeutic contexts. However, more investigation is required to determine its ideal use and comprehend the full scope of its advantages.

### 4.5 Viral hepatitis

The availability of secure and efficient direct antiviral treatments has restricted the promise of silymarin as a supportive therapy for viral hepatitis. However, several studies have suggested that silymarin may help those with acute or chronic hepatitis. This distinction is critical since silymarin is only approved for liver support, not for the direct treatment of viral hepatitis. Patients with cirrhosis or advanced fibrosis who had not responded to conventional therapy provided baseline information on silymarin use for the HALT‐C trial (Fengyun, 2012; Vincenzi et al., 2018). A total of 16% of the 1049 patients had taken silymarin in the past, with an average treatment lasting 6 months, and 16% had been taking it at the time of the baseline examination for an average of 35 months. Even though silymarin did not affect clinical outcomes, its usage was substantially linked to slower development of histologic liver pathology, as shown by reduced hepatic collagen concentration in biopsies when silymarin was being used (Hagag et al., 2016; Jiang et al., 2022).

In a different research, Egyptian patients with chronic hepatitis C were treated with either the commercial silymarin formulation (Eurosil 85®) or a low‐dose multivitamin supplement. At the 12‐month follow‐up, the fatigue symptoms and weight loss in both groups significantly improved from baseline. Patients who were given silymarin reported a considerable improvement in their symptoms of nausea and heartburn. Jaundice and dark urine were less frequent in the silymarin group, although the difference was not statistically significant. Except for social functioning and role emotions in the silymarin group and multivitamin group, most quality‐of‐life indicators significantly increased in both groups. Improvements in healthcare access and consistent nurse home visits made to both groups, which may not have been present in the trial community, may be responsible for the observed improvements in symptoms and quality of life (Ladas et al., 2010).

The Eurosil 85® formulation of silymarin showed a significant improvement in symptoms in an 8‐week randomized, placebo‐controlled study in Egyptian patients with acute viral hepatitis. When compared to patients who received a placebo, those who received silymarin saw biliary retention symptoms, such as dark urine, jaundice, and scleral icterus, resolve more quickly. Recipients of silymarin showed a significant drop in indirect bilirubin levels at day 56 as well. However, there were no appreciable differences in any hepatocellular damage indicators across the groups (Weiskirchen, 2016). Due to the availability of direct antiviral drugs, the use of silymarin for viral hepatitis has not gotten much attention; however, these results imply that silymarin may be useful in supportive therapy. To completely comprehend its particular benefits and restrictions in this situation, more research is required.

### 4.6 Safety and toxicity

When used as recommended and for a long time, silymarin has shown a fantastic safety profile in clinical studies. The suggested dosage of silymarin has been used in clinical trials for up to 48 weeks at a dose of 2100 mg/day and for up to 4 years at a dose of up to 420 mg/day. Overall, silymarin and silibinin side effects have been rather mild (Hagag et al., 2016). Clinical trial data have undergone systematic reviews that have revealed a low incidence of adverse events—rates of less than 4%, somewhat lower than those seen with a placebo. Notably, silymarin use has not been linked to any reports of fatalities or major adverse effects. Nearly 600 patients participated in placebo‐controlled trials, and just 0.68% of them discontinued their medication as a result of side effects, which was about the same as the placebo group's (0.67%) rate. Less than 1.5% of participants experienced the most frequently reported adverse effects, which were headaches and itching. Diarrhea, dyspepsia, irregular stools, and nausea were among the most frequently reported gastrointestinal adverse effects in open‐label trials with over 3500 individuals (Ladas et al., 2010). Less than 0.25% of patients experienced these situations, nevertheless.

On the safety of silymarin, especially in people with chronic hepatitis C, there has been much research. Patients were given varying doses of silymarin or a placebo for 7 days in a phase I randomized experiment. Mild to moderate nausea and a headache were temporary adverse effects that went away in a day and were not related to the medicine itself for one patient in the 240 mg group (Perumpail et al., 2017). In a related study, neither the silymarin group nor the multivitamin group had any patients stop their treatment due to side effects. These patients were Egyptians with chronic hepatitis C. Fatigue, headaches, diarrhea, and cramping/uncomfortable stomach were the most often mentioned adverse symptoms. Notably, there have never been any fatalities linked to side effects from silymarin use. A 57‐year‐old woman was described in a case report as experiencing intermittent sweating, nausea, colicky discomfort, diarrhea, vomiting, weakness, and collapse. Despite being the only serious adverse event to be reported, silymarin was thought to be possibly related to it (Sorrentino et al., 2015). Some patients taking milk thistle formulations have also mentioned mild laxative effects. When giving milk thistle to people who are known to be allergic to plants in the Asteraceae/Compositae family, caution should be used because isolated cases of anaphylactic reactions in patients have been documented.

## 5 RECENT STUDIES ON THE EFFICACY OF SILYMARIN

### 5.1 Antioxidant and anti‐inflammatory effects

A study by Rizzo et al. (2023) investigated the antioxidant capacity of silymarin in a murine model of NAFLD. The findings suggested that silymarin supplementation significantly reduced oxidative stress markers and inflammatory cytokine levels, supporting its potential as an adjunct therapy for NAFLD.

### 5.2 Impact on fibrosis and cirrhosis

In a randomized controlled trial conducted by Ibrahim et al. (2023), patients with early‐stage liver cirrhosis were administered silymarin for six months. The study reported a significant reduction in liver fibrosis markers and improvements in liver function tests, indicating a potential role for silymarin in preventing the progression of cirrhosis.

### 5.3 Combination therapies

Recent research by Mandal and Hazra (2023) explored the synergistic effects of silymarin when combined with conventional antiviral therapy in patients with hepatitis C. The study demonstrated enhanced virological response rates and improved liver function in the combination group, suggesting a promising avenue for adjunct therapies.

### 5.4 Global significance and future directions

The global prevalence of liver diseases necessitates the development of alternative treatments to complement existing therapeutic approaches. Silymarin, with its well‐documented safety profile and potential efficacy, holds promise as a supplementary or standalone option for liver disease management. Further research is warranted to establish standardized dosages, assess long‐term safety, and explore its effectiveness in diverse populations.

## 6 CONCLUSION

This review underscores the diverse potential of silymarin, a complex mixture with silybin as its primary component. It emphasizes the need to explore the individual components of silymarin to enhance scientific reproducibility and understand potential interactions between these compounds. The isolation of purified constituents is crucial for future research. Silymarin exhibits broad anti‐inflammatory, antioxidant, and pro‐apoptotic properties, affecting various cellular pathways and mechanisms. These attributes support its reported benefits in liver protection, neuroprotection, cardioprotection, and its potential as an anti‐cancer, anti‐viral, and anti‐diabetic agent, supported by extensive studies and experimental data. It has proven particularly effective in supporting liver function, reducing mortality due to liver disease, and preserving liver health by mitigating oxidative stress and cytotoxicity. Early intervention with silymarin treatment can maximize its therapeutic effects, making it a valuable complementary therapy for various liver conditions, including cirrhosis and liver damage induced by alcohol, drugs, and fatty liver disease.

## AUTHOR CONTRIBUTIONS

Hafiza Madiha Jaffar made substantial contributions in conception of work, manipulation and data acquisition. Faima Atta Khan, Fahad Al‐Asmari, and Muhammad Abdul Rahim conceptualized design of the work, data analysis, aided in drafting manuscript. Eliasse Zongo gave final approval of the version to be submitted.

## CONFLICT OF INTEREST STATEMENT

The authors declare no conflicts of interest.

## Data Availability

Data are contained within the article.
